# The clinical value of endoscopic ultrasonography combined with endoscopic treatment for upper gastrointestinal lifting lesions

**DOI:** 10.3389/fmed.2026.1765362

**Published:** 2026-05-05

**Authors:** Huanhuan Guo, Bo Peng, Zhang’e Xiong, Ziyin Li

**Affiliations:** Department of Gastroenterology, Wuhan Third Hospital, Guanggu Campus, Wuhan, Hubei, China

**Keywords:** endoscopic treatment, endoscopic ultrasonography, EUS assessments, stomach, upper gastrointestinal lifting lesions

## Abstract

**Objective:**

The present investigation is designed to assess the synergistic impact of endoscopic ultrasonography (EUS)paired with endoscopic therapy in addressing upper gastrointestinal (GI) lesions that protrude.

**Methods:**

Our approach involved retrospectively reviewing medical records to analyze 106 instances of upper GI protruding lesions identified via EUS at our institution, all of which were treated with either endoscopic techniques or surgery. Sample size was determined based on a prior pilot study, where the diagnostic concordance rate of EUS for upper GI protruding lesions was approximately75%. Using a margin of error of 5% and a confidence level of 95%, the calculated minimum sample size was83cases;106 patients were ultimately included to account for potential loss to follow-up. Comprehensive clinical and pathological information was systematically documented for each patient.

**Results:**

Among the106cases, endoscopic treatment was successful in101cases,2 cases were transferred to surgical treatment midway, and 3 cases were directly treated with surgery. The most common pathological types included polyps in 26 cases (24.53%), inflammatory changes in 25 cases (23.58%), leiomyomas in 22 cases (20.75%), gastrointestinal stromal tumors (GIST)in 11 cases (10.38%), and cysts in 10 cases (9.43%). The diagnostic concordance rate between EUS and pathological diagnosis was 81.13% (86/106; 95% confidence interval [CI]: 73.25–87.46%). For common lesion subtypes, the concordance rates were as follows: polyps (89.66%,26/29;95%CI:73.78–97.23%), leiomyomas (86.36%, 22/25; 95% CI: 68.17–95.84%), and GIST (73.33%, 11/15; 95% CI: 45.30–91.15%). Additionally, EUS showed favorable diagnostic performance for these lesions: sensitivity and specificity were 92.86%/98.04% for polyps,88.00%/97.06% for leiomyomas, and 73.33%/98.99% for GIST, respectively. Polyps are more common in the stomach and mostly originate from the mucosal layer; leiomyomas are more common in the esophagus and originate from the muscularis mucosae and the muscularis propria; GIST are more common in the stomach and mostly originate from the muscularis propria.

**Conclusion:**

The study’s conclusions highlight the remarkable precision of EUS in determining the intricacies of upper GI protrusions. Moreover, endoscopic treatments predicated on EUS assessments were found to be clinically safe, with a low incidence of postoperative adverse effects. Nonetheless, for rarer GI pathologies, such as schwannomas and granular cell tumors, there is an evident need for enhanced clinical acumen to mitigate the risk of misdiagnosis. The limitations of this single-center, retrospective study (e.g., small sample size, lack of long-term follow-up) should be considered when interpreting these findings.

## Introduction

The upper gastrointestinal (GI) tract presents a complex environment where protruding lesions create significant diagnostic difficulties (1–3). While traditional white-light endoscopy is crucial for initial assessments, it is inadequate for assessing the depth and characteristics of these complex lesions (4–7). Endoscopic ultrasonography (EUS) addresses this limitation by integrating endoscopic visualization with ultrasonic imaging, thereby overcoming the shortcomings of previous techniques (8–17). EUS allows for real-time examination of a lesion’s intraluminal profile, coupled with detailed ultrasonic imaging of the gut wall and surrounding structures, which improves diagnostic accuracy (18). This combined approach signifies a major advancement in the understanding and management of upper GI tract lesions, providing insights that were previously inaccessible.

Despite the recognized utility of EUS in evaluating upper GI protruding lesions, existing studies have critical gaps: (1) few have comprehensively correlated EUS findings with histopathology for rare lesion subtypes (e.g., schwannomas, granular cell tumors), leading to unclear diagnostic performance for these cases; (2) data on EUS-guided endoscopic treatment safety/efficacy are often limited by small single-center cohorts and lack statistical validation (e.g., confidence intervals, diagnostic performance metrics); and (3) standardized EUS interpretation protocols and inter-observer reliability assessments are rarely reported, undermining reproducibility. These gaps hinder the translation of EUS findings into standardized clinical practice.

This study retrospectively examines 106 patients with upper GI protruding lesions diagnosed via EUS and treated endoscopically or surgically at our institution. The primary goal is to evaluate the practical utility of EUS in diagnosing these lesions. By analyzing clinical, ultrasound, and pathological outcomes, we aim to contribute to the existing knowledge on EUS’s application and effectiveness in this specific context. This introduction lays the foundation for a thorough exploration of EUS’s role in managing upper GI protruding lesions, supported by a comprehensive review of clinical and pathological data from our patient cohort.

This study represents a significant advancement in gastrointestinal research, offering a novel perspective on the diagnostic management of upper GI protruding lesions. Its importance stems from the comprehensive retrospective analysis that connects EUS diagnostics with clinical outcomes, an area where, despite EUS’s recognized value, research remains limited. The novelty of this investigation lies in its detailed documentation and correlation of EUS findings with histopathological diagnoses, providing a solid basis for clinical decision-making. Additionally, this study pioneers the examination of EUS’s diagnostic accuracy for specific upper GI lesions, potentially reshaping treatment protocols and patient management strategies. By challenging conventional practices and offering new insights into EUS’s role in gastrointestinal diagnostics, this research contributes to the evolution of clinical practices, ultimately enhancing patient care and outcomes in gastroenterology.

## Materials and methods

### General data

A total of 106 patients with upper GI protruding lesions who underwent EUS examination and endoscopic or surgical treatment from January 2022 to June 2024 at Wuhan Third Hospital were included in this study, consisting of 51 males and 55 females, aged 24.0 to 73.0 years, with an average age of 52.12 years. Patients with severe coagulation disorders, acute upper GI bleeding, contraindications to anesthesia, or incomplete clinical/pathological data were excluded. Inter-observer reliability for EUS interpretation was assessed using Cohen’s kappa coefficient (*κ* = 0.82, indicating excellent agreement) between two gastroenterologists with >5 years of EUS experience (B.P. and Z.X.); disagreements were resolved by a third senior gastroenterologist with >10 years of experience. All patients were excluded from examination and treatment contraindications.

All patients underwent preoperative contrast-enhanced computed tomography (CT) of the abdomen to evaluate the extent of the lesions, their relationship with adjacent tissues and organs, and the presence of distant metastases, providing complementary information for treatment planning.

### Equipment and materials

The Olympus gastrointestinal endoscopy system CV-290 + CLV290SL and the Olympus Q260J, H260Z are utilized, along with the Olympus small probe UM-DP12-25R. For EUS, the OLYMPUS EU-ME1 type main unit and the GF-UCT240 linear array EUS are employed. In our institution’s endoscopic treatment arsenal, a cutting-edge German ERBE ICC-200 high-frequency electrosurgical unit is at the forefront, supported by a suite of specialized Olympus gear. This gear includes precise instruments such as injection needles for the precise delivery of medication, snares for the capture and excision of lesions, IT knives, and hook knives for the delicate cutting and dissection of tissues, and hot biopsy forceps that facilitate tissue sampling with minimal bleeding. Hemostatic clips are deployed to ensure hemostasis and manage any bleeding that may occur. CO₂ pumps and water pumps are also part of our toolkit, used to maintain the ideal conditions within the endoscopic field, which aids in procedure execution and improves visibility. This comprehensive selection of Olympus tools is carefully curated to support a broad spectrum of endoscopic procedures, with a focus on both effectiveness and patient safety.

### Treatment methods

Patients received venous anesthesia, with certain cases requiring additional tracheal intubation for anesthesia. The medical team closely monitored patients’ vital signs, including blood pressure, heart rate, breathing patterns, and blood oxygen levels, throughout the procedure. All endoscopic procedures (EMR, ESD, ESE, STER) were performed by operators with >3 years of experience in advanced endoscopic interventions, following the European Society of Gastrointestinal Endoscopy (ESGE) guidelines (12). The endoscopic approaches employed included EMR, ESD, ESE, and STER techniques to remove affected tissue. When surgical intervention was necessary, laparoscopic methods were utilized. Potential confounding variables (age, gender, comorbidities [hypertension, diabetes], lesion size) were collected to adjust for their impact on treatment outcomes.

All patients were scheduled for routine follow-up after treatment. The follow-up time points were set at 3, 6, and 12 months postoperatively. Follow-up was performed via outpatient revisit or telephone contact. At each follow-up, physical examination, routine blood tests, and upper gastrointestinal endoscopy were performed. Endoscopic ultrasonography (EUS) or abdominal enhanced computed tomography (CT) was selectively performed to evaluate local recurrence, residual lesions, and delayed complications such as bleeding, perforation, and stenosis.

## Results

### Localization and pathological characteristics of upper gastrointestinal protrusions

In a study of 106 instances of upper GI protrusions, the distribution was as follows: 34 cases in the esophagus, 63 in the stomach, and 9 in the duodenum. Post-treatment, the prevalence of common polyps was noted in 26 cases (24.53%), and inflammatory changes in 25 cases (23.58%), with a higher incidence in the stomach; leiomyomas were identified in 22 cases (20.75%), predominantly in the esophagus; GIST in 11 cases (10.38%), with a predilection for the stomach; and cysts in 10 cases (9.43%), predominantly in the esophagus, followed by the duodenum (Table 1).

**Table 1:** Distribution of lesions in upper gastrointestinal protrusions.

Site	Leiomyoma	GIST	Polyp	Cyst	Lipoma	Ectopic pancreas	Tumor	Inflammatory change	Neuroendocrine tumor	Granular cell tumor	Heterotopia of gastric mucosa	Schwannoma
Esophagus	17	0	2	7	0	0	1	5	0	1	1	0
Stomach	5	11	23	0	1	2	2	18	0	0	0	1
Duodenum	0	0	1	3	1	0	1	2	1	0	0	0

### Correlation between endosonographic layers and pathological findings

**Figure 1:**
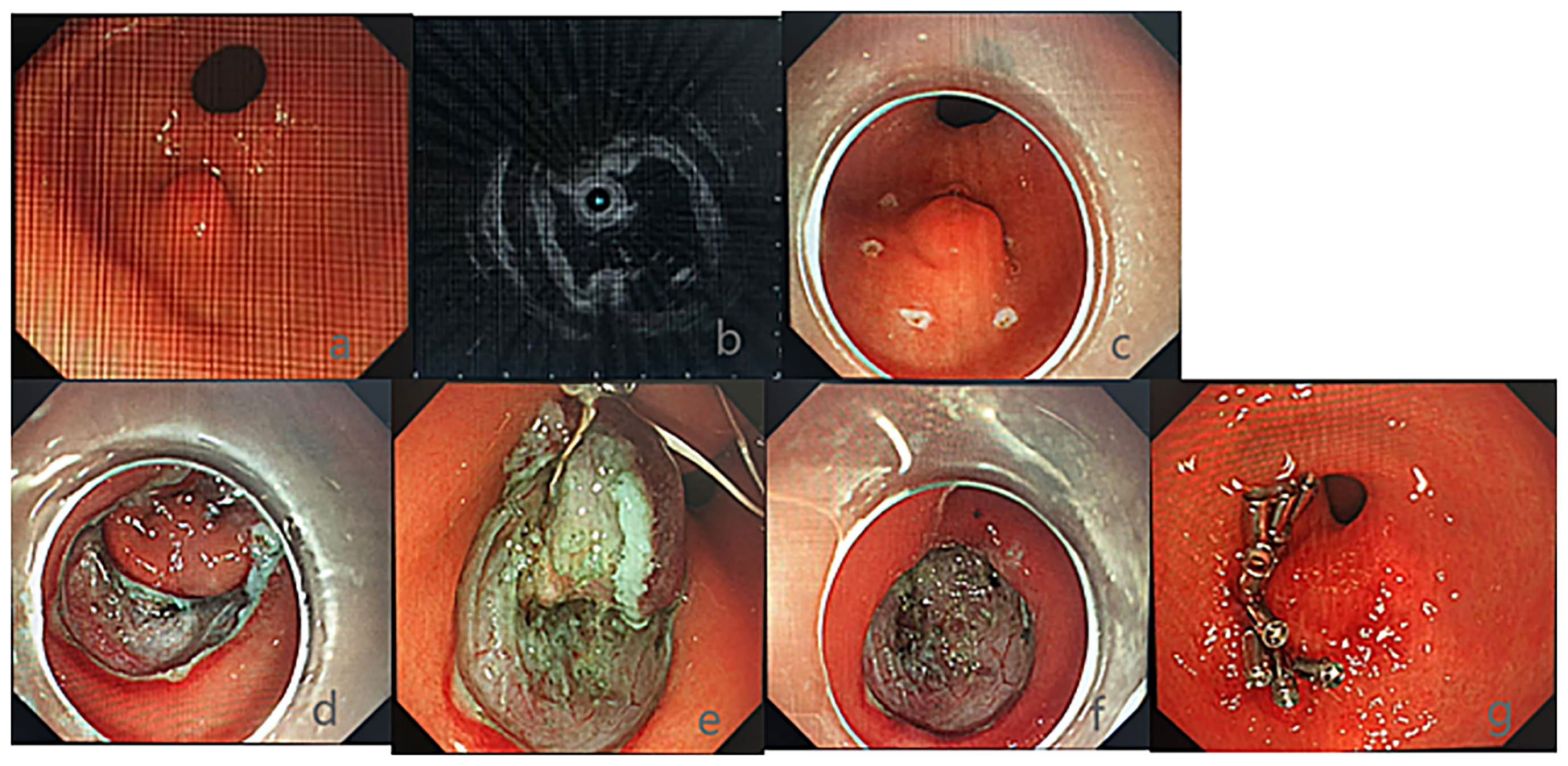
Endoscopic and ultrasonographic workflow for diagnosis and treatment of upper gastrointestinal submucosal lesions. Image brightness and contrast were uniformly adjusted using standard medical image editing software (e.g., Adobe Photoshop) to enhance clarity for publication; these adjustments did not alter the diagnostic features of the lesions. **(a)** Under white light endoscopy, a 0.8 cm submucosal protrusion is seen in the antrum of the stomach with a smooth mucosal surface, no ulceration, or hyperemia. **(b)** Endoscopic ultrasonography shows the lesion originating from the submucosal layer, with a hypoechoic, homogeneous texture and a visible tubular opening (arrow), suggestive of ectopic pancreas. **(c)** Marking the lesion boundary using a dual knife (Olympus KD-650L) at a 5-mm distance from the lesion edge to ensure complete resection. **(d–f)** Complete stripping of the lesion along the edge of the lesion with stepwise submucosal dissection; continuous hemostasis is performed using hot biopsy forceps (Olympus FB-25K-1) to prevent bleeding. **(g)** Metal clips (Olympus HX-610-135 L) closing the 1.0-cm mucosal defect to avoid perforation or delayed bleeding.

Endosonographic analysis indicated that among lesions originating from the mucosal layer, polyps, inflammatory changes, and cysts were frequently detected, with 23 cases (21.7%), 19 cases (17.92%), and 6 cases (5.66%) respectively. In the muscularis mucosae, leiomyomas were the most common, accounting for 16 cases (15.09%). Ectopic pancreas and lipomas were each found in 2 cases (1.89%) within the submucosal layer. In the muscularis propria, GIST were the predominant finding, with 11 cases (10.38%), followed by 5 cases of leiomyomas and 1 case of schwannoma (Figure 1; Table 2).

**Table 2:** Relationship between EUS layer and pathological diagnosis.

EUS layer	Lesion type	Number of cases (*n*)	Percentage (%)
Mucosal layer	Polyp	23	21.70%
	Inflammatory change	19	17.92%
	Cyst	6	5.66%
	Tumor	1	0.94%
	Leiomyoma	1	0.94%
	Gastric mucosa heterotopia	1	0.94%
	Neuroendocrine tumor	1	0.94%
Muscularis mucosae layer	Leiomyoma	16	15.09%
	Inflammatory change	5	4.72%
	Cyst	3	2.83%
	Polyp	1	0.94%
Submucosal layer	Ectopic pancreas	2	1.89%
	Lipoma	2	1.89%
	Adenocarcinoma	2	1.89%
	Polyp	2	1.89%
	Granular cell tumor	1	0.94%
	Cyst	1	0.94%
	Inflammatory change	1	0.94%
Muscularis propria layer	Gastrointestinal stromal tumor (GIST)	11	10.38%
	Leiomyoma	5	4.72%
	Schwannoma	1	0.94%

### Concordance rate of endoscopic ultrasonography and pathological diagnosis

In the 106 cases assessed with EUS, the breakdown was as follows: 29 cases (27.36%) of polyps, 25 cases (23.58%) of leiomyomas, 15 cases (14.15%) of GIST, 10 cases (9.43%) of cysts, 4 cases (3.77%) of ectopic pancreas, 4 cases (3.77%) of tumors, and 2 cases (1.89%) of lipomas, along with individual cases of neuroendocrine tumors and fat streaks. Eighty-six cases aligned with pathological outcomes, yielding a diagnostic concordance rate of 81.13% (86/106). As detailed in Table 3, the concordance rates for polyps, leiomyomas, and GIST were 89.66% (26/29; 95% CI: 73.78–97.23%), 86.36% (22/25; 95% CI: 68.17–95.84%), and 73.33% (11/15; 95% CI: 45.30–91.15%), respectively. EUS also demonstrated high specificity for these lesions (≥97%), though sensitivity varied (73.33% for GIST vs. 92.86% for polyps) (Table 3). The concordance rates for the more frequent polyps, leiomyomas, and GIST with pathology were 89.66% (26/29), 86.36% (22/25), and 73.33% (11/15) respectively (Table 3). Discrepancies between EUS and pathological diagnosis for rare lesions are presented in Table 4. The main misclassification events included 3 cases of cysts misdiagnosed as leiomyomas, 1 case of schwannoma misdiagnosed as GIST, and 1 case of granular cell tumor misdiagnosed as lipid plaque (Table 4). In addition, EUS overdiagnosed inflammatory changes in 10 cases and showed false-positive results for gastric mucosa heterotopia and lipid plaques, respectively (Table 4).

**Table 3:** Diagnostic performance of EUS for common upper gastrointestinal protruding lesions.

Lesion type	Pathology (*n*)	EUS (*n*)	Concordance rate (%, 95 CI)	Sensitivity (%, 95 CI)	Specificity (%, 95 CI)
Polyps	26	29	89.66 (73.78–97.23)	92.86 (76.98–99.11)	98.04 (89.68–99.97)
Leiomyomas	22	25	86.36 (68.17–95.84)	88.00 (69.79–97.21)	97.06 (90.07–99.60)
GIST	11	15	73.33 (45.30–91.15)	73.33 (45.30–91.15)	98.99 (94.92–99.96)

**Table 4:** Discrepancies between EUS and pathological diagnosis for rare lesions.

Lesion type	Pathology (*n*)	EUS diagnosis (*n*)	Misdiagnosed cases (*n*)	Misclassification
Cysts	10	10	3	Misdiagnosed as leiomyomas
Inflammatory changes	25	15	10	Overdiagnosed by EUS
Schwannomas	1	0	1	Misdiagnosed as GIST
Granular cell tumors	1	0	1	Misdiagnosed as lipid plaque
Gastric mucosa heterotopia	0	1	1	False positive
Lipid plaques	0	1	1	False positive

### Clinical treatment outcomes

Among the 106 patients, endoscopic treatment was effective in 101 cases. Two cases required conversion to surgical treatment during the endoscopic procedure, and three cases were directly escalated to surgery. No complications such as gastrointestinal bleeding, infection, or perforation were observed in patients who underwent endoscopic treatment. The two patients who required conversion had lesions in the gastric body, measuring approximately 2.4 cm and 4 cm in maximum diameter, originating from the muscularis propria and preliminarily identified as GIST. These cases involved abundant blood vessels, making tumor resection and incision highly susceptible to bleeding, which was deemed intractable via endoscopy. After consultation with the patients’ families, they were transferred to surgical treatment. Pathological examination confirmed one case as a low-risk GIST and the other as a schwannoma. Among the three patients directly transferred to surgery, one had a duodenal mass with preoperative linear ultrasonography suggesting the involvement of the lower common bile duct and pancreatic duct, rendering endoscopic treatment unsuitable. Another patient had lesions in the gastric fundus, antrum, and ascending colon, originating from the muscularis propria and preoperatively identified as multiple GIST, leading to direct surgical intervention. Pathological examination revealed a gastric GIST and a schwannoma in the ascending colon. The third case involved a mucosal protrusion based on a residual stomach, suspected to be a tumor with focal submucosal layer involvement, and surgical treatment was chosen after considering the clinical history and the family’s wishes.

Among the 106 patients, 102 patients completed follow-up and 4 patients were lost to follow-up, with a loss to follow-up rate of 3.77%. The median follow-up time was 8 months (range: 2–16 months). During the follow-up period, no local recurrence, residual lesions, or delayed complications (including delayed bleeding, perforation, and anastomotic stenosis) were detected by endoscopy, EUS, or abdominal enhanced CT.

## Discussion

This study confirms that EUS-guided endoscopic therapy achieves not only a high success rate (98.06%, 101/103) and low complication rate, but more importantly, provides critical information for clinical decision-making by accurately determining the layer of origin and lesion characteristics. This precision effectively avoids unnecessary surgical interventions, thereby reducing medical costs, shortening hospital stays, and promoting faster patient recovery while achieving minimally invasive treatment.

Elevated lesions are commonly observed during gastroscopy, and conventional gastroscopy can only assess their size and shape, making further judgment of the lesions challenging. EUS can observe the origin level, size, internal echo, margins, blood flow, and the relationship with adjacent structures of the lesions, which is highly valuable for judging submucosal lesions (19, 20). Most studies indicate that EUS reports on submucosal lesions are mostly benign, with 13% being malignant and 8% potentially malignant (21), making EUS examination crucial for guiding clinical treatment options.

Our findings confirm EUS’s high diagnostic value for upper GI protruding lesions, with an overall concordance rate of 81.13% (95% CI: 73.25–87.46%). This is consistent with the 78–85% range reported in prior domestic studies (22). Compared with a recent multicenter study from Korea (23) that reported a concordance rate of 83.7% for gastric subepithelial tumors, our findings are comparable, supporting the generalizability of EUS diagnostic performance across different clinical settings. For specific lesion subtypes, the concordance rate for polyps (89.66%) and leiomyomas (86.36%) in our cohort aligns with values reported by Lei et al. (22) (87.2 and 84.6%, respectively). For GIST, the concordance rate (73.33%) was slightly lower than that reported in some Asian multicenter studies (23) (78.9%), which may reflect differences in case mix and the higher proportion of small GISTs in our series where overlapping features with leiomyomas are more pronounced.

The study demonstrated that, among EUS layers and distributions, polyps tended to be more prevalent in the mucosal layer, mainly in 23 respective instances in the stomach; leiomyomas were mainly located in the muscularis mucosae and muscularis propria of the esophagus; GIST mostly originated from the muscularis propria in the stomach (11/11); and cysts were mainly present in the mucosa and muscularis mucosae, most predominantly observed in the esophagus.

Misdiagnoses in this study occurred primarily in three scenarios. First, among lesions originating from the muscularis mucosae, three esophageal cysts were misclassified as leiomyomas. Cysts typically appear as anechoic lesions with clear boundaries on EUS, but when located in non-submucosal layers, they may lose typical anechoic characteristics and present as hypoechoic masses, leading to confusion with leiomyomas (24). To reduce such diagnostic errors, when a well-defined hypoechoic lesion is identified in the muscularis mucosae or muscularis propria, careful attention should be paid to the homogeneity of the internal echo. The presence of subtle anechoic areas, combined with endoscopic features such as translucency or a soft texture upon gentle probing, should raise suspicion of a cyst. If diagnostic uncertainty persists, fine-needle aspiration (FNA) may be considered for confirmation.

Second, among lesions originating from the muscularis propria, one gastric schwannoma was misdiagnosed as GIST. Both tumors commonly present as hypoechoic, well-defined masses arising from the muscularis propria, with overlapping EUS features (25). When a lesion originates from the muscularis propria and presents as a well-defined, homogeneous hypoechoic mass on EUS, schwannoma should be included in the differential diagnosis. Schwannomas often exhibit a characteristic hyperechoic rim (“edge halo”), and their clinical prognosis differs from that of GIST. Preoperative endoscopic ultrasound-guided fine-needle aspiration (EUS-FNA) is crucial for pathological differentiation.

Third, one esophageal granular cell tumor was misdiagnosed as a lipid plaque. Granular cell tumors are rare in the digestive tract, with 5–9% occurring in the esophagus, and typically originate from the mucosal propria layer, appearing as molar-like protrusions under endoscopy. The rarity of these entities likely contributed to the diagnostic errors, highlighting the need for enhanced clinical acumen to mitigate the risk of misdiagnosis.

Treatment outcomes confirmed the safety of EUS-guided endoscopic therapy: 98.06% success rate (101/103) and no major complications (bleeding, perforation), which aligns with Lei et al. (22). Of the 103 patients who underwent endoscopic treatment, two cases were transferred to surgery for laparoscopic intervention during the procedure, with a success rate of 98.06% (101/103), and no gastrointestinal major bleeding, infection, perforation, or other serious complications occurred postoperatively. It can be seen that endoscopic treatment after EUS assessment is safe and effective, consistent with literature reports (22). The two patients who were transferred to surgery had lesions with diameters greater than 2 cm, and preoperative EUS assessment indicated abundant blood flow within the lesions. It can be seen that larger lesions with abundant blood flow lead to repeated bleeding during resection and prolonged treatment time, which may be the reason for transferring to surgery, indicating that clinicians need to be more cautious with endoscopic treatment for such cases.

This study has notable limitations. First, it is a single-center, retrospective study with potential selection bias (e.g., exclusion of patients with incomplete data). Second, the small sample size (*n* = 106) limits statistical power for rare lesions (e.g., schwannoma, granular cell tumor). Third, the lack of long-term follow-up prevents evaluation of recurrence rates and long-term efficacy. Fourth, no comparison with other diagnostic modalities (e.g., contrast-enhanced CT) limits assessment of EUS’s relative value. Additionally, EUS has inherent limitations in assessing larger lesions (e.g., maximum diameter exceeding 4–5 cm). The limited penetration depth of ultrasound may prevent complete and accurate evaluation of the full extent of the lesion and its relationship with surrounding structures, a factor that should be considered in treatment decision-making. Future studies should adopt a multi-center, prospective design with larger cohorts and include ≥12 months of follow-up to address these gaps. Incorporating predictive models, such as that proposed by Kim et al. (23), may further improve the differentiation between GIST and leiomyomas.

## Conclusion

In summary, EUS has high accuracy in determining the level and nature of elevated lesions in the upper GI tract, with a high concordance rate with pathology, and endoscopic treatment after assessment is safe with low postoperative adverse reactions. However, EUS still has a certain misdiagnosis rate for lesions originating from the muscular layer. Clinicians need continuous learning and practice for such lesions and can combine imaging or clinical data for comprehensive judgment, to reduce clinical misdiagnosis rates, reduce unnecessary treatment and medical resource consumption, and provide the best clinical treatment plans for patients.

## Data Availability

The raw data supporting the conclusions of this article will be made available by the authors, without undue reservation.
